# Possible Role of Aurora-C in Meiosis

**DOI:** 10.3389/fonc.2015.00178

**Published:** 2015-08-13

**Authors:** Kuo-Tai Yang, Chieh-Ju C. Tang, Tang K. Tang

**Affiliations:** ^1^Department of Animal Science and Technology, National Taiwan University, Taipei, Taiwan; ^2^Institute of Biomedical Sciences, Academia Sinica, Taipei, Taiwan

**Keywords:** meiosis, oocyte, spermatocyte, aurora kinase, mitosis, polyploidy, male infertility, aneuploidy

## Abstract

The meiotic generation of haploid gametes with equal contents of genetic material is important for sexual reproduction in mammals. Errors in the transmission of chromosomes during meiosis may lead to aneuploidy, which is the leading cause of miscarriage and congenital birth defects in humans. The Aurora kinases, which include Aurora-A, Aurora-B, and Aurora-C, are highly conserved serine–threonine kinases that play essential roles in centrosome function, chromosome segregation, and cytokinesis during mitosis and meiosis. While Aurora-A and Aurora-B have been extensively studied in mitosis, the role of Aurora-C in meiosis is only now starting to be revealed. For example, the perturbation of Aurora-C kinase activity by microinjection of Aurora-C-kinase-dead mutant mRNAs into mouse oocytes induced multiple defects, including chromosome misalignment, abnormal kinetochore–microtubule attachment, premature chromosome segregation, and failure of cytokinesis during meiotic division. However, the analysis of such defects is complicated by the possibility that Aurora-B may be present in mammalian germ cells. Interestingly, a homozygous mutation of Aurora-C in humans leads to the production of large-headed polyploid spermatozoa and causes male infertility, but homozygous females are fertile. Mouse studies regarding the roles of Aurora-B and Aurora-C in female meiotic divisions have yielded inconsistent results, and it has proven difficult to explain why homozygous human females have no significant clinical phenotype. In this review, we will discuss the controversial status of Aurora-B in oocytes and the possible role of Aurora-C during meiotic division.

## Introduction

An essential process during the sexual reproduction of mammals is the production of haploid gametes from diploid precursors. This is done via meiosis, which consists of a single round of DNA duplication and two rounds of cell division that are called meiosis I (MI) and meiosis II (MII). Homologous chromosomes are segregated in MI, while sister chromatids are separated in MII via a process similar to that seen during mitosis (1, 2). Failures in chromosome segregation at meiosis result in aneuploidy, which is a major cause of miscarriages and birth defects in humans. However, the mechanisms underlying such failures are not completely understood (3). The Aurora kinases belong to a family of serine/threonine kinases that are pivotal in the regulation of cell division processes, including mitosis (4, 5) and meiosis (6–8). There are three Aurora kinases in mammals: Aurora-A and Aurora-B are ubiquitously expressed, and their functional roles in mitosis have been extensively studied (9–11); whereas Aurora-C is mainly restricted to germ cells (12), and is beginning to be functionally studied in meiosis. It is interesting to note that these three kinases share sequence homology in their central catalytic kinase domains, but differ widely in their N- and C-terminal sequences (12). Mouse Aurora-B and Aurora-C share 77.6% amino acid sequence identity in their catalytic domains, while Aurora-A and Aurora-C share only 66.3% sequence identity in this region, suggesting that there may be a close functional link between Aurora-B and -C (12).

Aurora-C (also called AIE1/AIE2/STK13) was first identified in the Tang lab, in a screening for kinases expressed in sperm and eggs (12), and also independently by Bernard et al. in a homologous kinase screening in a human placental cDNA library (13). Aurora-A and -B are ubiquitously expressed in many tissues, particularly in actively dividing cells. In contrast, Aurora-C is predominantly expressed in the testis (12, 13) and is mainly restricted to meiotically active germ cells, including spermatocytes (14) and oocytes (6). Aurora-C was reported to be overexpressed in a variety of human cancer cell lines (15, 16) and ectopic overexpression of Aurora-C can also induce cell transformation and tumor formation (17). However, its expression in tumor cells and normal somatic tissues is still the matter of some debate (14, 18). Aurora-B is a member of the chromosomal passenger complex (CPC), which localizes to the centromeres/kinetochores from prophase to metaphase and to the central spindle and midbody during cytokinesis (19, 20). In contrast, endogenous Aurora-C protein has never been detected in normal somatic cells by immunofluorescence or Western blot analyses using fully validated antibodies (6, 14). Instead, ectopically expressed tagged Aurora-C has been detected in transfected cells, where it showed a localization pattern similar to that of Aurora-B (21–23). The role of Aurora-B in meiotic chromosome orientation during meiosis has recently been reviewed by Watanabe (1). In this review, we will focus on the possible role of Aurora-C during male and female meiotic divisions.

## Aurora-C in Mouse Spermatocytes: Subcellular Localization, Transcriptional Regulation, and Functional Implications

**Figure 1:**
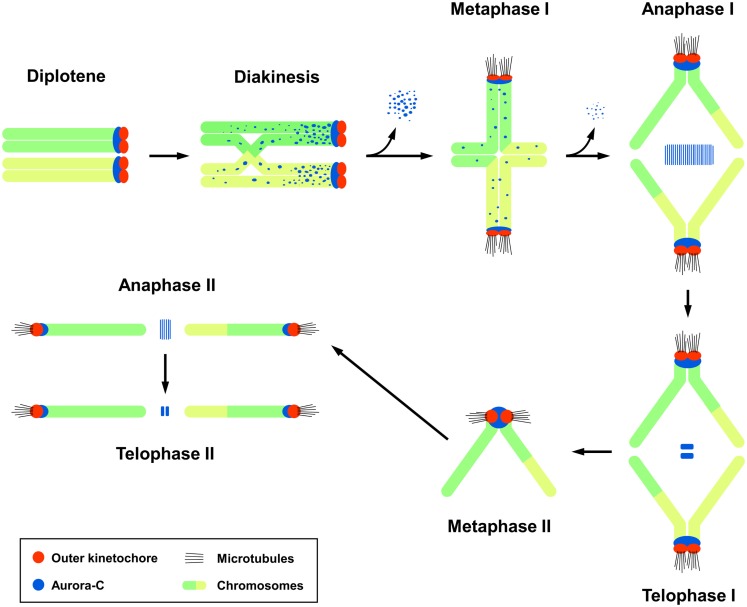
**The subcellular localization of Aurora-C during male mouse meiosis**. Aurora-C is labeled dark blue and outer kinetochores are labeled red. Chromosomes are labeled green/yellow. Figure modified from Ref. (14). Aurora-B shows a similar localization pattern as that of Aurora-C (24). Aurora-C signals were gradually lost from the chromosome arms and accumulated at the centromeres during the diakinesis-to-metaphase I transition (14). Currently, it is not clear whether Aurora-B also targets to the chromosome arms in diakinesis chromosomes. INCENP was first detected at the zygotene (24), prior to the appearance of Aurora-B and -C, and co-localized with Aurora-B (24) and Aurora-C at a later diplotene stage (14). INCENP has been implicated to recruit Aurora-C (14) and Aurora-B (24) to meiotic chromosomes.

The subcellular localization of endogenous Aurora-C during male meiotic division had been carefully examined by confocal immunofluorescence microscopy in mouse spermatocytes (14). In germ cells, the meiotic prophase consists of five sequential stages: leptotene, zygotene, pachytene, diplotene, and diakinesis. Aurora-C was first detected at the centromeric regions in early diplotene spermatocytes, after which it was found to spread along the chromosomal arms of sister chromatids during diakinesis. Upon the transition from diakinesis to MI, Aurora-C gradually dissociates from the chromosome arms and becomes concentrated at the centromeres near the kinetochores. Thereafter, it relocalizes to the spindle midzone and midbody during the anaphase I/telophase I and anaphase II/telophase II transitions, respectively (Figure 1) (14). A similar localization pattern was reported for Aurora-B in mouse spermatocytes (14, 24). However, while Aurora-B was detected in mitotic spermatogonia, Aurora-C was not, suggesting that Aurora-C may play a unique role in male meiotic division (14).

The finding that Aurora-C and -B co-localize during male meiotic divisions raised several interesting questions: (i) how are Aurora-C/-B recruited to the appropriate positions to execute their meiotic functions during spermatogenesis? (ii) Do Aurora-C/-B play similar or different roles during male meiotic divisions? (iii) Since Aurora-C is mainly restricted in germ cells, how is Aurora-C regulated during spermatogenesis?

In somatic cells, Aurora-B is a member of the CPC along with several non-enzymatic subunits, including INCENP, survivin, and Borealin; together, the members of this complex contribute to regulation of chromosome segregation, microtubule–kinetochore attachments, and cytokinesis (19, 25). INCENP contains a conserved C-terminal IN-box that binds Aurora-B (26) and an N-terminal region that targets to centromeres (27).

Interestingly, INCENP can be detected in meiotic cells prior to the appearances of Aurora-B and -C (14, 24). It is first found at the central element (CE) of the synaptonemal complex (SC), from the zygotene to late pachytene stages (24). It then moves to heterochromatic chromocenters (14, 24) and co-localizes with Aurora-B and -C at the diplotene stage (14). Immunoprecipitation analyses showed that INCENP can form distinct complexes with either Aurora-C (INCENP/Aurora-C) or Aurora-B (INCENP/Aurora-B) in the testis (14). Together, these findings strongly support a model, in which INCENP recruits Aurora-C and -B to their appropriate locations and activates them to execute their meiotic functions in spermatocytes (14). Consistent with this notion, INCENP was reported to bind (21, 22) and activate Aurora-C (21) in somatic cells, and ectopically expressed Aurora-C was found to associate with survivin (28) and borealin (29). However, the functional linkage of these proteins during meiotic divisions has not yet been fully resolved. Recent studies have shown that BUB1, shugoshin proteins, and haspin kinase are also required for targeting Aurora-B to the centromeres of meiotic chromosomes (30–34). It will be interesting to test whether these proteins are also required for Aurora-C targeting to the centromeres in the future.

What is the role of Aurora-B and -C during male meiotic divisions? In somatic cell mitosis, Aurora-B and Polo-like kinase 1 (Plk1) phosphorylate the cohesion complexes to promote their dissociation from the chromosome arms (35–37). Interestingly, during meiosis, some SC components (e.g., SCP2 and SCP3) and cohesion subunits (e.g., SMC1b and SMC3, but not REC8) are gradually released from the chromosome arms and accumulate at the centromeres during the prophase I to metaphase I transition (38, 39). In accordance with this finding, Aurora-C was reported to be dissociated from the chromosome arms and concentrated at the centromeres during the diakinesis–metaphase I transition (14). Together, this seems to suggest that Aurora-C might regulate the release of cohesion subunits and SC components from the chromosome arms during MI. Future work is needed to test this possibility.

To investigate the role of Aurora-B/-C in spermatogenesis, Kimmins et al. (40) generated transgenic mice in which a pachytene-specific promoter drove the expression of an inactive Aurora-B mutant, and produced *Aurora-C* knockout mice by homologous recombination. Expression of the inactive Aurora-B dominant-negative (DN) mutant severely impaired spermatogenesis, resulting in abnormal spermatocytes, increased apoptosis, and spermatogenic arrest. The *Aurora-C* null mice were viable and had normal testis weights, sperm counts, and meiotic progression, but some of the mutant males were sterile and had sperm abnormalities, including heterogeneous chromatin condensation, loose acrosomes, and blunted heads (40). As Aurora-B (24) and Aurora-C (14) co-localize and associate with INCENP, it has proven difficult to differentiate their roles in spermatogenesis. Furthermore, it is unclear why *Aurora-C* null mice show only minor sperm-related alterations. Previous reports have shown that ectopic expression of an Aurora-C kinase-dead mutant disrupts the association of INCENP with Aurora-B (22) and that Aurora-C can complement the function of Aurora-B Kinase in somatic cells (21, 23, 41). Thus, it is possible that endogenous Aurora-B could compensate for the function of Aurora-C in the *Aurora-C* null mice and that ectopic expression of the Aurora-B DN mutant could non-specifically block the function of endogenous Aurora-C in *Aurora-B* mutant mice. Alternatively, studies have suggested that multiple tandem copies of the *Aurora-C* gene (42) or a potential “functional pseudogene” in the mouse genome may alleviate the spermatogenic effects in the *Aurora-C* null mice. Thus, why do mammals require both Aurora-C and -B kinases in spermatocytes? Do they play overlapping or differential roles during male meiotic divisions? These questions remain open in the context of mammalian spermatocytes.

Finally, the transcriptional regulation of Aurora-C during spermatogenesis is poorly understood. Our group isolated the cDNA clones encoding human TZFP (testis zinc finger protein) and mouse Tzfp, which are predominantly expressed in testis (43, 44). Human TZFP and mouse Tzfp contain a conserved N-terminal BTB (bric-a-brac, tramtrack, broad complex)/POZ (poxvirus, zinc finger) domain and three C-terminal C2H2 zinc fingers (43, 44). Interestingly, the zinc finger domain of TZFP/Tzfp is closely related to the promyelocytic leukemia zinc finger (PLZF) protein, a known DNA-binding transcriptional repressor (45). Biochemical studies demonstrated that the C-terminal zinc finger domain of Tzfp directly binds to the TGTACAGTGT motif (designated as the Tzfp binding site, or tbs), located in the upstream flanking sequence of the *Aurora-C/Aie1* gene (44). These studies also showed that the N-terminal BTB/POZ domain has repressor activity, suggesting that Tzfp may negatively regulate *Aurora-C* gene expression in spermatocytes (44). Consistent with this notion, Tzfp is highly expressed in spermatocytes at the pachytene stage in MI, and *Tzfp*-knockout mice show downregulation of *Aurora-C/Aie1* expression (46).

## Aurora-C/-B in Mouse Oocytes: Subcellular Localization and Potential Functions during Female Meiotic Divisions

**Figure 2:**
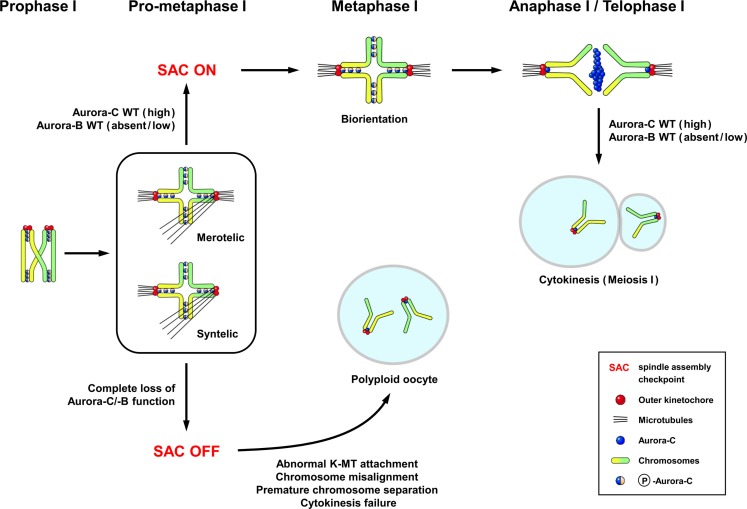
**The subcellular localization of Aurora-C and its possible functions in female mouse meiosis I**. The localization pattern of Aurora-C in oocytes (6) is similar to that reported in spermatocytes (14). Aurora-C is phosphorylated at Thr171 and located at the chromosome axes and centromeres during late prophase–metaphase I. Aurora-C is dephosphorylated and relocalized to the midzone and midbody during anaphase I–telophase I transition (6). Endogenous Aurora-B appears to be either undetectable (6) or present at low levels in mouse (49) and human oocytes (50). Complete loss of both Aurora-C and Aurora-B activities by ectopic expression of Aurora-C kinase-dead mutant caused more severe effects, including chromosome misalignment, aberrant kinetochore–microtubule (K-MT) attachments, premature chromosome segregation, and cytokinesis failure in meiosis I, resulted in producing polyploid oocytes (6, 7). Figure modified from Ref. (6).

The localization of endogenous Aurora-C has been examined in detail during the various stages of meiotic division in mouse oocytes (6). Aurora-C was detected at the chromosome axes and centromeres in prometaphase I–metaphase I, in which Aurora-C was also phosphorylated at Thr171 (Figure 2) (6). During the anaphase I–telophase I transition, Aurora-C was dephosphorylated and relocalized to the midzone and midbody (Figure 2) (6), and thus shows a pattern similar to that reported in spermatocytes (14). Interestingly, protein kinase A (PKA) can phosphorylate recombinant Aurora-C/Aie1 protein *in vitro* at Thr171 (47), yet its physiological meaning is not clear. Unexpectedly, no endogenous Aurora-B protein was detected on the meiotic chromosomes of mouse oocytes when assessed by immunofluorescence staining with the same antibody that had successfully detected Aurora-B in spermatocytes (6) nor was it detected in experiments using other antibodies and fixation conditions (48). In contrast, Balboula and Schindler (7) detected endogenous Aurora-B at the nuclei of prophase-arrested oocytes and the meiotic spindle at metaphase I and metaphase II. This apparent discrepancy may reflect the specificities of the utilized different antibodies or other, yet unknown factors.

In experiments using exogenous proteins, GFP-Aurora-B expressed in injected oocytes was clearly detected at the centromeres/kinetochores at metaphase I (6, 48, 49, 51) and at the spindle midzone and midbody during the anaphase I–telophase I transition (6, 48, 51), thereby showing a pattern similar to that of endogenous Aurora-C (6). Furthermore, it was reported that *Aurora-C* mRNA is recruited for translation more efficiently than the *Aurora-B* mRNA, and that exogenously expressed Aurora-B protein is not stable during meiosis (49). Thus, despite the abundance of the mRNAs for Aurora-B and Aurora-C in mouse oocytes (6, 49) and the high-level expression of the Aurora-C protein in both male and female mouse germ cells (6, 14), little or no Aurora-B protein appears to be expressed in mouse oocytes. This interesting observation suggests that the translation of Aurora-B protein level is differentially regulated in female germ cells.

The role of Aurora-C in oocytes has recently been investigated using a number of approaches, including exogenously expressed Aurora-C kinase-dead or gatekeeper mutants (6, 7), treatment with small molecule inhibitors (ZM447439 and AZD1152) of Aurora kinases (6, 48, 51, 52), siRNA-mediated knockdown (51), and the generation of *Aurora-C* knockout (*Aurkc^−/−^*) mice (7, 49, 53). Yang et al. (6) first reported that exogenous expression of kinase-dead Aurora-C mutant (T171A, T175A, designated Aurora-C-KD) in mouse oocytes significantly inhibited endogenous Aurora-C activity and produced multiple defects, including chromosome misalignment, abnormal kinetochore–microtubule (K-MT) attachment, premature chromosome segregation, and failure of cytokinesis in MI. This phenotype was partially recapitulated in oocytes injected with an INCENP-targeting siRNA (51), in an INCNEP-delIN deletion mutant that lacked the Aurora-C-binding motif (6), and in oocytes treated with high doses of small molecule inhibitors of Aurora-B (ZM447439 and AZD1152), that are also likely to inhibit Aurora-C (6, 51, 52). Unexpectedly, *Aurkc^−/−^* knockout mice were found to be subfertile (49). The overall percentage of chromosome misalignment in MI oocytes of *Aurkc^−/−^* mice was not strikingly different from that of wild-type controls, but a portion of the oocytes in knockout mice arrested in MI and displayed abnormally aligned chromosomes (49). Recently, Balboula and Schindler (7) developed an ATP-binding-pocket-Aurora-C mutant (L93A, gatekeeper mutant) that appears to selectively disrupt the function of Aurora-C, but not Aurora-B, during female meiotic divisions, and microinjected this mutant into mouse oocytes. Their observations suggested that the specific loss of Aurora-C function caused chromosome misalignment and failure to correct erroneous K–MT attachments (7), which is similar to the deficits observed in oocytes expressing the Aurora-C kinase-dead mutant (T171A/T175A) (6). Meanwhile, the process of cytokinesis in oocytes appears to be regulated by either the Aurora-B–CPC complex or by the activities of both Aurora-B and Aurora-C (7).

In sum, there is currently no suitable model that encompasses all of the reported roles of Aurora-C during female meiotic divisions. The efforts to generate such a consensus have been complicated by the possible functional compensation of Aurora-B in oocytes (7, 48, 49, 51, 54), the lack of selectivity and specificity among the known small molecule inhibitors (6, 51, 52), problems with the efficiency of siRNA knockdown (51), and the possible presences of multiple tandem copies of the *Aurora-C* gene (42) and/or a potential “functional pseudogene” in the mouse genome. Given these limitations, however, the speculated roles of Aurora-C and -B during female meiotic divisions are summarized in Figure 2.

## Aurora-C/-B in Human Germ Cells and Preimplantation Embryos: Subcellular Localization and Aurora-C-Deficient Human Patients

Recently, Santos et al. (50) reported the localizations and mRNA expression levels of endogenous Aurora-B and Aurora-C in human germ cells and preimplantation embryos developed from tri-pronuclear (3PN) zygotes. They observed the signal corresponding to Aurora-C in the region surrounding the centromeres in human MI and MII oocytes. This was consistent with the localization pattern described in mouse oocytes (6). Human Aurora-C first appeared at the pericentric heterochromatin in pachytene spermatocytes (50), whereas mouse Aurora-C was first detected at the diplotene stage (6). In contrast, endogenous Aurora-B was hardly detected in human oocytes at MI (50).

In preimplantation embryos, Aurora-C appears to be the major Aurora kinase expressed during the first three embryonic cell cycles, where it can be visualized on prometaphase chromosomes in zygotes and two- and four-cell-stage human embryos. The endogenous Aurora-B protein was expressed at low-to-undetectable levels during these embryonic stages, but increased significantly after the eight-cell stage. It is interesting to note that the expression of Aurora-C occurs earlier, and is completely replaced by Aurora-B at the blastocyst stage of human embryonic development. These findings prompted the authors to hypothesize that Aurora-C could be the main enzymatic component of the CPC, and thus plays a specific role during human female meiosis and preimplantation embryo development (50). However, it is not yet clear whether its deficiency is linked to a high aneuploidy rate in human preimplantation embryos.

Recently, three naturally occurring mutations in the human *Aurora-C* kinase gene were reported to be associated with male infertility: c.144delC, which deletes a cytosine in exon 3 (8); c.686G > A, which is a missense mutation in exon 6 (p.Cys229Tyr) (55); and c.436-2A.G, which is a splicing site mutation that leads to the skipping of exon 5 (56). Individual males carrying homo- or hetero-allelic combinations of null or strong loss-of-function Aurora-C mutations frequently produce polyploidy and multi-flagellar spermatozoa that are unsuitable for fertilization. Males homozygous for c.144delC had no obvious physiological or anatomical defects beyond sperm abnormalities, suggesting that Aurora-C is not essential for somatic cell division (55). Moreover, females carrying the same homozygous mutation (c.144delC) were fertile, suggesting that Aurora-C may be dispensable for meiotic divisions in the human female (55).

The question of how the large-headed multi-flagellar polyploid spermatozoa are generated in humans cannot be answered using the *Aurkc^−/−^* knockout mice. However, speculations can be made. One possible explanation is that Aurora-C plays a critical role in cytokinesis during spermatogenesis. Indeed, mouse oocytes injected with Aurora-C-kinase-dead mRNAs showed failure in the cytokinesis of MI (6). This resulted in the production of large polyploid mouse oocytes, which could be compared to the polyploid spermatocytes found in Aurora-C-deficient humans. However, we cannot yet explain why Aurora-C-deficient human females are fertile and do not have polyploid oocytes.

## Conclusion

In mouse spermatocytes, both Aurora-B (24) and Aurora-C (14) proteins are present at relatively high levels and show a similar localization pattern (Figure 1). Both are also likely to be recruited to meiotic chromosomes by INCENP (14, 24). The functional differences in these proteins during male meiotic divisions remain largely unknown. In females, endogenous Aurora-B is either undetectable (6) or present at low levels in mouse (49) and human oocytes (50). Here, Aurora-C appears to be the major enzymatic component of the CPC, and thus may play a specific role during female meiotic divisions (6, 49–51). The differential roles of Aurora-B and Aurora-C during female meiosis have been addressed by a number of different approaches, but no conclusive answer has yet been obtained. Furthermore, it is difficult to use the results obtained from mouse studies to interpret the clinical phenotypes in human Aurora-C-deficient subjects. For example, microinjection of Aurora-C-kinase-dead mRNAs into mouse oocytes caused failure of cytokinesis in MI and the production of large polyploid oocytes (6), whereas a homozygous Aurora-C mutation in human affects male (but not female) germ cells. This discrepancy could reflect species-specific differences, and further studies are needed to resolve the differential roles of Aurora-B and Aurora-C during meiotic divisions in mouse and human germ cells.

## Conflict of Interest Statement

The authors declare that the research was conducted in the absence of any commercial or financial relationships that could be construed as a potential conflict of interest.

## References

[1] Watanabe Y. Geometry and force behind kinetochore orientation: lessons from meiosis. Nat Rev Mol Cell Biol (2012) 13:370–82. 10.1038/nrm3349 22588367

[2] Duro E Marston AL. From equator to pole: splitting chromosomes in mitosis and meiosis. Genes Dev (2015) 29:109–22. 10.1101/gad.255554.114 25593304 PMC4298131

[3] Hassold T Hall H Hunt P. The origin of human aneuploidy: where we have been, where we are going. Hum Mol Genet (2007) 16(R2):R203–8. 10.1093/hmg/ddm243 17911163

[4] Glover DM Leibowitz MH McLean DA Parry H. Mutations in aurora prevent centrosome separation leading to the formation of monopolar spindles. Cell (1995) 81:95–105. 10.1016/0092-8674(95)90374-7 7720077

[5] Andrews PD Knatko E Moore WJ Swedlow JR. Mitotic mechanics: the auroras come into view. Curr Opin Cell Biol (2003) 15:672–83. 10.1016/j.ceb.2003.10.013 14644191

[6] Yang KT Li SK Chang CC Tang CJ Lin YN Lee SC Aurora-C kinase deficiency causes cytokinesis failure in meiosis I and production of large polyploid oocytes in mice. Mol Biol Cell (2010) 21:2371–83. 10.1091/mbc.E10-02-0170 20484572 PMC2903667

[7] Balboula AZ Schindler K. Selective disruption of Aurora C kinase reveals distinct functions from Aurora B kinase during meiosis in mouse oocytes. PLoS Genet (2014) 10:e1004194. 10.1371/journal.pgen.1004194 24586209 PMC3937256

[8] Dieterich K Soto Rifo R Faure AK Hennebicq S Ben Amar B Zahi M Homozygous mutation of AURKC yields large-headed polyploid spermatozoa and causes male infertility. Nat Genet (2007) 39:661–5. 10.1038/ng2027 17435757

[9] Carmena M Ruchaud S Earnshaw WC. Making the Auroras glow: regulation of Aurora A and B kinase function by interacting proteins. Curr Opin Cell Biol (2009) 21:796–805. 10.1016/j.ceb.2009.09.008 19836940 PMC2806521

[10] Hochegger H Hegarat N Pereira-Leal JB. Aurora at the pole and equator: overlapping functions of Aurora kinases in the mitotic spindle. Open Biol (2013) 3:120185. 10.1098/rsob.120185 23516109 PMC3718339

[11] Meraldi P Honda R Nigg EA. Aurora kinases link chromosome segregation and cell division to cancer susceptibility. Curr Opin Genet Dev (2004) 14:29–36. 10.1016/j.gde.2003.11.006 15108802

[12] Tseng TC Chen SH Hsu YP Tang TK. Protein kinase profile of sperm and eggs: cloning and characterization of two novel testis-specific protein kinases (AIE1, AIE2) related to yeast and fly chromosome segregation regulators. DNA Cell Biol (1998) 17:823–33. 9809744 10.1089/dna.1998.17.823

[13] Bernard M Sanseau P Henry C Couturier A Prigent C. Cloning of STK13, a third human protein kinase related to Drosophila aurora and budding yeast Ipl1 that maps on chromosome 19q13.3-ter. Genomics (1998) 53:406–9. 10.1006/geno.1998.5522 9799611

[14] Tang CJ Lin CY Tang TK. Dynamic localization and functional implications of Aurora-C kinase during male mouse meiosis. Dev Biol (2006) 290:398–410. 10.1016/j.ydbio.2005.11.036 16386730

[15] Tsou JH Chang KC Chang-Liao PY Yang ST Lee CT Chen YP Aberrantly expressed AURKC enhances the transformation and tumourigenicity of epithelial cells. J Pathol (2011) 225:243–54. 10.1002/path.2934 21710690

[16] Zekri A Lesan V Ghaffari SH Tabrizi MH Modarressi MH. Gene amplification and overexpression of Aurora-C in breast and prostate cancer cell lines. Oncol Res (2012) 20:241–50. 10.3727/096504013X13589503482978 23581231

[17] Khan J Ezan F Cremet JY Fautrel A Gilot D Lambert M Overexpression of active Aurora-C kinase results in cell transformation and tumour formation. PLoS One (2011) 6:e26512. 10.1371/journal.pone.0026512 22046298 PMC3203144

[18] Keen N Taylor S. Aurora-kinase inhibitors as anticancer agents. Nat Rev Cancer (2004) 4:927–36. 10.1038/nrc1502 15573114

[19] Ruchaud S Carmena M Earnshaw WC. Chromosomal passengers: conducting cell division. Nat Rev Mol Cell Biol (2007) 8:798–812. 10.1038/nrm2257 17848966

[20] Carmena M Earnshaw WC The cellular geography of aurora kinases. Nat Rev Mol Cell Biol (2003) 4:842–54. 10.1038/nrm1245 14625535

[21] Li X Sakashita G Matsuzaki H Sugimoto K Kimura K Hanaoka F Direct association with inner centromere protein (INCENP) activates the novel chromosomal passenger protein, Aurora-C. J Biol Chem (2004) 279:47201–11. 10.1074/jbc.M403029200 15316025

[22] Chen HL Tang CJ Chen CY Tang TK. Overexpression of an Aurora-C kinase-deficient mutant disrupts the Aurora-B/INCENP complex and induces polyploidy. J Biomed Sci (2005) 12:297–310. 10.1007/s11373-005-0980-0 15917996

[23] Sasai K Katayama H Stenoien DL Fujii S Honda R Kimura M Aurora-C kinase is a novel chromosomal passenger protein that can complement Aurora-B kinase function in mitotic cells. Cell Motil Cytoskeleton (2004) 59:249–63. 10.1002/cm.20039 15499654

[24] Parra MT Viera A Gomez R Page J Carmena M Earnshaw WC Dynamic relocalization of the chromosomal passenger complex proteins inner centromere protein (INCENP) and aurora-B kinase during male mouse meiosis. J Cell Sci (2003) 116:961–74. 10.1242/jcs.00330 12584241

[25] Kelly AE Funabiki H. Correcting aberrant kinetochore microtubule attachments: an Aurora B-centric view. Curr Opin Cell Biol (2009) 21:51–8. 10.1016/j.ceb.2009.01.004 19185479 PMC2801027

[26] Honda R Korner R Nigg EA. Exploring the functional interactions between Aurora B, INCENP, and survivin in mitosis. Mol Biol Cell (2003) 14:3325–41. 10.1091/mbc.E02-11-0769 12925766 PMC181570

[27] Ainsztein AM Kandels-Lewis SE Mackay AM Earnshaw WC. INCENP centromere and spindle targeting: identification of essential conserved motifs and involvement of heterochromatin protein HP1. J Cell Biol (1998) 143:1763–74. 10.1083/jcb.143.7.1763 9864353 PMC2175214

[28] Yan X Cao L Li Q Wu Y Zhang H Saiyin H Aurora C is directly associated with survivin and required for cytokinesis. Genes Cells (2005) 10:617–26. 10.1111/j.1365-2443.2005.00863.x 15938719

[29] Slattery SD Moore RV Brinkley BR Hall RM. Aurora-C and Aurora-B share phosphorylation and regulation of CENP-A and borealin during mitosis. Cell Cycle (2008) 7:787–95. 10.4161/cc.7.6.5563 18239465

[30] Kawashima SA Tsukahara T Langegger M Hauf S Kitajima TS Watanabe Y. Shugoshin enables tension-generating attachment of kinetochores by loading Aurora to centromeres. Genes Dev (2007) 21:420–35. 10.1101/gad.1497307 17322402 PMC1804331

[31] Kawashima SA Yamagishi Y Honda T Ishiguro K Watanabe Y. Phosphorylation of H2A by Bub1 prevents chromosomal instability through localizing shugoshin. Science (2010) 327:172–7. 10.1126/science.1180189 19965387

[32] Kelly AE Ghenoiu C Xue JZ Zierhut C Kimura H Funabiki H. Survivin reads phosphorylated histone H3 threonine 3 to activate the mitotic kinase Aurora B. Science (2010) 330:235–9. 10.1126/science.1189505 20705815 PMC3177562

[33] Wang F Dai J Daum JR Niedzialkowska E Banerjee B Stukenberg PT Histone H3 Thr-3 phosphorylation by Haspin positions Aurora B at centromeres in mitosis. Science (2010) 330:231–5. 10.1126/science.1189435 20705812 PMC2967368

[34] Yamagishi Y Honda T Tanno Y Watanabe Y. Two histone marks establish the inner centromere and chromosome bi-orientation. Science (2010) 330:239–43. 10.1126/science.1194498 20929775

[35] Losada A Hirano M Hirano T. Cohesin release is required for sister chromatid resolution, but not for condensin-mediated compaction, at the onset of mitosis. Genes Dev (2002) 16:3004–16. 10.1101/gad.249202 12464631 PMC187494

[36] Sumara I Vorlaufer E Stukenberg PT Kelm O Redemann N Nigg EA The dissociation of cohesin from chromosomes in prophase is regulated by polo-like kinase. Mol Cell (2002) 9:515–25. 10.1016/S1097-2765(02)00473-2 11931760

[37] Waizenegger IC Hauf S Meinke A Peters JM. Two distinct pathways remove mammalian cohesin from chromosome arms in prophase and from centromeres in anaphase. Cell (2000) 103:399–410. 10.1016/S0092-8674(00)00132-X 11081627

[38] Lee J Iwai T Yokota T Yamashita M. Temporally and spatially selective loss of Rec8 protein from meiotic chromosomes during mammalian meiosis. J Cell Sci (2003) 116:2781–90. 10.1242/jcs.00495 12759374

[39] Eijpe M Offenberg H Jessberger R Revenkova E Heyting C. Meiotic cohesin REC8 marks the axial elements of rat synaptonemal complexes before cohesins SMC1beta and SMC3. J Cell Biol (2003) 160:657–70. 10.1083/jcb.200212080 12615909 PMC2173354

[40] Kimmins S Crosio C Kotaja N Hirayama J Monaco L Hoog C Differential functions of the Aurora-B and Aurora-C kinases in mammalian spermatogenesis. Mol Endocrinol (2007) 21:726–39. 10.1210/me.2006-0332 17192404

[41] Slattery SD Mancini MA Brinkley BR Hall RM. Aurora-C kinase supports mitotic progression in the absence of Aurora-B. Cell Cycle (2009) 8:2984–94. 10.4161/cc.8.18.9591 19713763

[42] Hu HM Chuang CK Lee MJ Tseng TC Tang TK. Genomic organization, expression, and chromosome localization of a third aurora-related kinase gene, Aie1. DNA Cell Biol (2000) 19:679–88. 10.1089/10445490050199063 11098217

[43] Lin W Lai CH Tang CJ Huang CJ Tang TK. Identification and gene structure of a novel human PLZF-related transcription factor gene, TZFP. Biochem Biophys Res Comm (1999) 264:789–95. 10.1006/bbrc.1999.1594 10544010

[44] Tang CJ Chuang CK Hu HM Tang TK. The zinc finger domain of Tzfp binds to the tbs motif located at the upstream flanking region of the Aie1 (aurora-C) kinase gene. J Biol Chem (2001) 276:19631–9. 10.1074/jbc.M100170200 11279021

[45] Li X Lopez-Guisa JM Ninan N Weiner EJ Rauscher FJ III Marmorstein R. Overexpression, purification, characterization, and crystallization of the BTB/POZ domain from the PLZF oncoprotein. J Biol Chem (1997) 272:27324–9. 10.1074/jbc.272.43.27324 9341182

[46] Furu K Klungland A. Tzfp represses the androgen receptor in mouse testis. PLoS One (2013) 8:e62314. 10.1371/journal.pone.0062314 23634227 PMC3636255

[47] Chen SH Tang TK. Mutational analysis of the phosphorylation sites of the Aie1 (Aurora-C) kinase in vitro. DNA Cell Biol (2002) 21:41–6. 10.1089/10445490252810302 11879579

[48] Shuda K Schindler K Ma J Schultz RM Donovan PJ. Aurora kinase B modulates chromosome alignment in mouse oocytes. Mol Reprod Dev (2009) 76:1094–105. 10.1002/mrd.21075 19565641 PMC3187553

[49] Schindler K Davydenko O Fram B Lampson MA Schultz RM. Maternally recruited Aurora C kinase is more stable than Aurora B to support mouse oocyte maturation and early development. Proc Natl Acad Sci U S A (2012) 109:E2215–22. 10.1073/pnas.1120517109 22778418 PMC3421190

[50] Santos MA van de Werken C de Vries M Jahr H Vromans MJM Laven JSE A role for Aurora C in the chromosomal passenger complex during human preimplantation embryo development. Hum Reprod (2011) 26:1868–81. 10.1093/humrep/der111 21493633

[51] Sharif B Na J Lykke-Hartmann K McLaughlin SH Laue E Glover DM The chromosome passenger complex is required for fidelity of chromosome transmission and cytokinesis in meiosis of mouse oocytes. J Cell Sci (2010) 123:4292–300. 10.1242/jcs.067447 21123620 PMC2995614

[52] Swain JE Ding J Wu J Smith GD. Regulation of spindle and chromatin dynamics during early and late stages of oocyte maturation by aurora kinases. Mol Hum Reprod (2008) 14:291–9. 10.1093/molehr/gan015 18353803 PMC2408935

[53] Fernandez-Miranda G Trakala M Martin J Escobar B Gonzalez A Ghyselinck NB Genetic disruption of Aurora B uncovers an essential role for Aurora C during early mammalian development. Development (2011) 138:2661–72. 10.1242/dev.066381 21613325

[54] Yoshida S Kaido M Kitajima TS. Inherent instability of correct kinetochore-microtubule attachments during meiosis I in oocytes. Dev Cell (2015) 33:589–602. 10.1016/j.devcel.2015.04.020 26028219

[55] Dieterich K Zouari R Harbuz R Vialard F Martinez D Bellayou H The Aurora Kinase C c.144delC mutation causes meiosis I arrest in men and is frequent in the North African population. Hum Mol Genet (2009) 18:1301–9. 10.1093/hmg/ddp029 19147683

[56] Ben Khelifa M Zouari R Harbuz R Halouani L Arnoult C Lunardi J A new AURKC mutation causing macrozoospermia: implications for human spermatogenesis and clinical diagnosis. Mol Hum Reprod (2011) 17:762–8. 10.1093/molehr/gar050 21733974 PMC3639514

